# Alphalinolenic acid, a potent inhibitor of fatty acid synthase antimycobacterial agent

**DOI:** 10.1186/1471-2334-14-S3-E19

**Published:** 2014-05-27

**Authors:** Mallika Jainu, A Hemaprabha, S Vinutha, Sarah Rajitha, TS Ranjani

**Affiliations:** 1 Department of Biomedical Engineering, Sri Siva Subramaniya Nadar College of Engineering, Kalavakkam, Chennai -603110, India

## Background

The mycobacteria become resistant since their slow growth phase increases its ability to adapt and acquire new resistance mechanisms. Presently the first line therapy against TB includes the use of isoniazid and rifampin, which are probably the most effective mycobacterial drugs available today and then there is a very small chance to treat the disease since other newer drugs are seriously limited by gastrointestinal, renal and/or neurological toxicities. Therefore, the search for new ways to treat TB is of primary importance and urgency. Alphalinolenic acid (ALA) is a potent inhibitor of fatty acid synthase (FAS) in a variety of prokaryotic and eukaryotic cells.

## Methods

Using a standardized mycobacterial susceptibility test, we have observed that ALA inhibits the growth of several species of mycobacteria, including tuberculous species such as *Mycobacterium tuberculosis* (H37Rv and clinical isolates) and *Mycobacterium bovis* BCG.

## Results

Alpha linolenic acids are toxic to mycobacteria, such as *Mycobacterium tuberculosis* H37Rv, with minimum inhibitory concentrations (MICs) of 25-50 μg/mL and *Mycobacterium bovis* with minimum inhibitory concentration of 6.25 – 25 μg/mL.

## Conclusion

Our data demonstrate that ALA as a prototypical inhibitor, significant antimycobactericidal effects was seen with *Mycobacterium tuberculosis* and *Mycobacterium bovis*. A possible antibacterial mechanism was postulated to proceed via disruption of the bacterial cell membrane resulting in a change in membrane permeability.

